# Evaluation of peripheral neuropathy in lower limbs of patients with rheumatoid arthritis and its relation to fall risk

**DOI:** 10.1186/s42358-022-00238-3

**Published:** 2022-03-22

**Authors:** Fabio de Araújo Pereira, Mariana de Almeida Lourenço, Marcos Renato de Assis

**Affiliations:** 1 Neurology Department, Marilia Medical School (FAMEMA), 800 Monte Carmelo Avenue, Marília, SP 17519030 Brazil; 2 Physiotheraphy Departament, São Paulo State University (UNESP), 737 Hygino Muzzi Filho Avenue, Marília, SP 17525-900 Brazil; 3 Rheumathology Department, Marilia Medical School (FAMEMA), 800 Monte Carmelo Avenue, Marília, SP 17519030 Brazil

**Keywords:** Rheumatoid arthritis, Peripheral nervous system diseases, Accidental falls, Postural balance

## Abstract

**Background:**

Rheumatoid Arthritis (RA) is a chronic disabling systemic disease characterized by joint inflammation, and extra-articular manifestations, including peripheral neuropathy, a condition that can be associated with changes in muscle strength, proprioception and postural balance contributing for the risk of falls. The objective of this study is to analyze the incidence of peripheral neuropathy in patients with RA and its association with the occurrence of falls.

**Methods:**

Patients were assessed by an electroneuromyography (ENMG) exam and by a questionnaire on accidental falls occurrence in the previous 12 months. They were also assessed on balance by the Short Physical Performance Battery (SPPB), functionality by the Health Assessment Questionnaire (HAQ), disease activity by the Disease Activity Score (DAS-28), neuropathic pain by the Questionnaire for the Diagnosis of Neuropathic Pain (DN4), and cutaneous sensitivity of the feet by the monofilament testing of Semmes–Weinstein. Monthly calls on falls were made in the subsequent six months. Data analysis was performed using the Shapiro–Wilk test for normality and Spearman, Chi-square, and T-student correlation tests, with a significant P level ≤ 0.05.

**Results:**

A sample of 33 patients were evaluated. The incidence of peripheral neuropathy was 48.5%, of which 68.7% were axonal and 31.3% myelinic. The sensorimotor type was present in 64.7%, motor in 17.6%, and sensorial in 11.7% of the cases. Neuropathy was associated to balance (*P* = 0.026), neuropathic pain (*P* = 0.016), deep tendon reflexes absence (*P* = 0,049), altered skin sensitivity of the feet (*P* = 0.029) and fear of falling (*P* = 0.001). No association was found between peripheral neuropathy and age, gender, disease activity, or functionality. No significant association was found between peripheral neuropathy and occurrence of falls, in a 12-month retrospective and 6-month prospective evaluation.

**Conclusion:**

Peripheral neuropathy has a high incidence in patients with RA, and is related to neuropathic pain, altered postural balance, but not to the occurrence of falls.

## Background

Rheumatoid arthritis (RA) is a chronic systemic autoimmune disabling disease in which synovial inflammation leads to a deforming symmetrical polyarthritis. Its prevalence in adults ranges from 0.4 to 1.3% of the general population [1–3].

Since the 1990s studies have shown a high risk of falls in patients with RA compared to the general population, varying between 10 and 54% in these patients [4–6]. In the elderly population well-defined predictors to the occurrence of falls have been classified in biological, behavioral, environmental, and socioeconomic factors [7]. In RA, studies have associated some conditions, such as previous history of falling in the last year, polypharmacy, use of antidepressants, psychotropic drugs, number of comorbidities, number of painful and swollen joints, pain intensity and static balance alteration. However the evidence is limited based in only one study with conflicting results to other studies [4].

Peripheral neuropathy often shortened to neuropathy, is a general term describing damage to the neuronal cells and fibers with variable causes, including compression, toxic, genetic, metabolic, and connective tissue diseases [8, 9]. Neuropathy has been identified as an important risk factor for falls in the elderly population [10]. In RA, Hart et al. [11] began investigating the relationship between peripheral neuropathy and RA, but only in 1965, Good et al. [12], performed the first study using Electroneuromyography (ENMG) to access neuropathy in RA, showing a high incidence of neuropathy that is often subclinical. Later studies have confirmed neuropathy is frequent in RA patients and sometimes could require an ENMG exam to differentiate symptoms related to joint inflammation or neuropathy [13–16].

A correlation between neuropathy in lower limbs and altered balance is well documented as an important risk factor for falls in the general population, but no study has investigated this correlation in RA patients [7, 17–19].

The objective of this study is to evaluate the relationship between neuropathy in lower limbs, balance, and the occurrence of falls in RA patients.

## Methods

This is a cross-sectional study with Rheumatoid Arthritis outpatients from Marilia Medical School. A 6-months follow-up on falls were also performed.

Participants were selected by convenience from patients diagnosed with RA in the rheumatology outpatient clinic, by invitation during consultation or by telephone call.

Patients with rheumatoid arthritis according to the classification criteria for RA 2010 ACR/EULAR and 18 years of age or older were included, and those with cognitive impairment, lower limb amputation, uncontrolled hypothyroidism, amyloidosis, alcoholism, and renal failure on dialysis treatment were excluded.

The sample size was estimated to test the association between peripheral neuropathy and falls in patients with RA assuming a large effect size (0.50) according to the study by Richardson et al. [10], with a study power of 80%, a type I margin of error of 5%, and one degree of freedom, resulting in the minimum sample size of 32 individuals. The sample size calculation was performed in the software G*Power, version 3.1.9.2 (Franz Faul, Universität Kiel, Germany).

Data collection was performed in the outpatient clinic from December 2019 until January 2021. After signing the Informed Consent Form, patients were submitted to questionnaires, physical examination, and clinical tests.

The patients were submitted to a neurological physical examination with assessment of deep tendon reflexes, vibratory sensitivity, and assessment of the cutaneous sensitivity of the feet with Semmes–Weinstein monofilament, and then the ENMG examination was performed.

Blood samples were collected in the same day and were analyzed at the blood center to perform the erythrocyte sedimentation rate and C-reactive protein tests.

In order to minimize information bias, the assessment of disease activity (DAS-28) was performed in all patients by the same rheumatologist, and all other tests and questionnaires and examination were performed by the same neurologist.

To minimize the recall bias, a 6 months prospective evaluation was performed with monthly phone contact questioning about falls. The calls were made by trained physicians.

### Assessment instruments

Disease activity was assessed by the Disease Activity Score (DAS-28), Simplified Disease Activity Index (SDAI) and Clinical Disease Activity Index (CDAI); functionality was assessed by the Health Assessment Questionnaire (HAQ) [20, 21]; neuropathic pain by the Questionnaire for Diagnosis of Neuropathic Pain (DN4) [22]; and balance by the Short Physical Performance Battery—SPPB [23].

### Clinical neurological examination

Patellar and Achilles deep tendon reflexes was performed bilaterally with Babinski’s hammer, and vibratory sensitivity was examined on the lateral knee prominences and malleolus bilaterally with a 128 Hz tuning fork, according to standard neurological semiology.

**Fig. 1:**
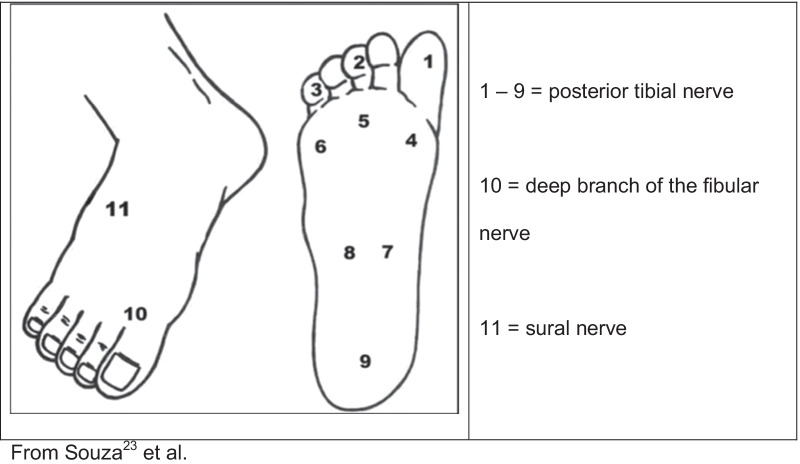
Visual representation of the cutaneous points for sensitivity assessment. From Souza et al. [23]

Skin sensitivity was examined with Semmes–Weinstein monofilament [24] on specific territories as shown in Fig. 1.

Neurophysiological evaluation by electroneuromyography test was performed with a NIHON-KOHDEN MEB-9400 K (Tokyo, Japan) device in the lower limbs. Sural nerve sensory conduction, and fibular and tibial nerves motor conduction were analyzed by the Quantitative EMG software QP-946BK (Tokyo, Japan). Skin temperature was maintained between 31 and 34 °C. Sensory conduction was studied antidromically on the sural nerve bilaterally.

Motor conduction in the tibial nerve was studied through orthodromic stimulation in the ankle and popliteal fossa, and uptake in the abductor hallucis brevis muscle, including F-waves. Orthodromic motor conduction was studied on the fibular nerve with stimulation at the ankle and below the head of the fibula, and uptake in the extensor digitorum brevis muscle, including F-wave. All amplitudes were determined on a base-to-peak value basis. Peak latency and onset latency were measured for the sensory nerve action potential (SNAP) and compound motor action potential (CMAP), respectively. The conduction velocity of each nerve was measured. Myography was performed with a concentric needle, assessing insertion activity, resting activity, and response to spontaneous contraction in the medial gastrocnemius, tibialis anterior, rectus femoris, and extensor digitorum brevis muscles bilaterally. Neuropathy was determined according to the parameters defined by Preston [25].

The fall occurrence was assessed by a semi-structured questionnaire, previously approved, and used by this group to study RA patients. The number and the characterization of the falls in the previous 12 months were recorded. Monthly phone calls were made in the following six months to prospectively assess the occurrence of falls.

### Statistical analysis

The comparison between two independent groups was performed by the Student’s t-test for unpaired samples based on the homogeneity of variances observed in Levene's test.

The association between qualitative variables was analyzed by the Chi-square association test. The Odds ratio for falling was calculated and analyzed using the 95% confidence interval (95%CI). The normality distribution was analyzed by the Shapiro–Wilk test.

The correlation between number of falls, fear of falling and SPPB with the study variables was performed by Spearman's test. The quantitative variables that showed significant correlation were represented in the scatter plot and analyzed by the linear R2, which represents the explanation factor, indicating the percentage of variation of the dependent variable (y-axis) explained by the variation of the independent variable (x-axis).

SPSS software version 19.0 for Windows was used, with a significance level of 5%.

## Results

**Fig. 2:**
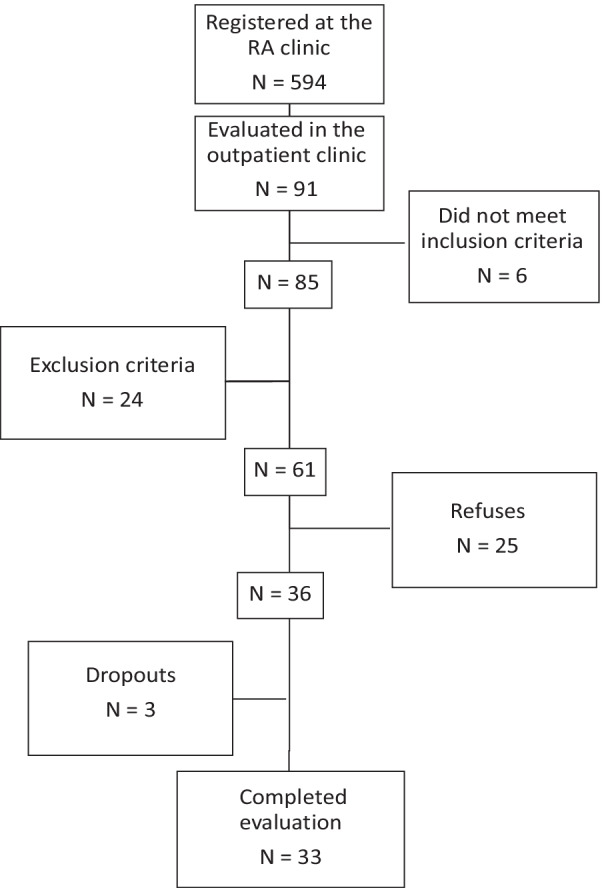
Flowchart of sample selection

The complete evaluation was performed in 33 RA patients and the selection of participants is shown in Fig. 2.

The excluded patients were, 7 for dementia, 12 for alcoholism, 3 for dialysis, and 2 for amputation of lower limbs. There was a high number of refusals to the ENMG exam due to the possibility of unpleasant symptoms related to electric shocks and needle insertion. There where tree dropouts, two caused by refusals and one compromised by a high degree of obesity compromising exam quality. ENMG after having performed the other tests. The sample characteristics are described in Table 1.

**Table 1:** Rheumatoid arthritis patients sample characteristic

Variable	Value
*Gender (%)*	
Female	84.8
Male	15.5
*Age (years)*	
Mean (SD)	59.8 (9.4)
Min.–Máx	36–77
*BMI (kg/m*^*2*^*)*	
Mean (SD)	27.4 (6.1)
Mín.–Máx	16.8–41.2
*Medications (number of)*	
Median	6
Mín.–Máx	2–12
*Schooling (%)*	
No schooling	3
Elementary school (incomplete/complete)	30.3
Middle school (incomplete/complete)	30.3
High school (incomplete/complete)	33.3
Higher education (incomplete/complete)	3
*Marital status (%)*	
Single	18.2
Married	54.5
Divorced	24.2
Widow(er)	3.1
*Ethnicity (%)*	
White	51.5
Brown	21.2
Black	27.3
*Disease duration (years)*	
Median	11
Min.–Máx	1–44
*Disease activity*	
DAS-28_ESR_	
Median	3.42
Mín.–Máx	1.74–7.53
DAS-28_CRP_	
Median	2.53
Mín.–Máx	1.36–6.15
CDAI	
Median	5
Mín.–Máx	0–43
SDAI	
Median	12.2
Mín.–Máx	1.3–112.7
*Functionality (HAQ)*	
Median	1
Mín.–Máx	0–2.25
*Comorbities (%)*	
Tipe 2 diabetes	21.2
Tipe 1 diabetes	3
Arterial hipertension	54.5
Hypothyroidism	21.2
Depression	18.1

SD, standard deviation; Min, minimum; Max, maximum; Kg, kilogram; BMI, Body Mass Index; DAS-28_ESR_, Disease Activity Score-28—erythrocyte sedimentation rate; HAQ, Health Assessment Score

Among the patients, 23 were using immunobiological disease course modifying drugs (DCMD) and only 4 were using corticosteroids at the time of the evaluation. The use of benzodiazepines was reported only by 4 patients and antidepressants by 9.

Peripheral neuropathy was found in 48.5% of the patients and the characteristics of the changes in ENMG are described in Table 2.

**Table 2:** Electroneuromyography examination

Neuropathy	Number of affected	%
*Injury type*		
Axonal	11	68.7
Mielinic	5	31.3
*Affected nerve type*		
Sensory	2	12.5
Motor	3	18.7
Sensorimotor	11	68.7

Deep reflexes were absent in 42.4% of the cases, and 81.8% and 40.7% among them reported pain in the lower limbs, classified as neuropathic pain.

Reduced sensitivity was found in the area corresponding to the sural nerve in 72.7% of the patients, fibular in 69.6%, and posterior tibial nerve in 72.7%. The associations of peripheral neuropathy to the main variables are shown in Table 3.

**Table 3:** Main outcomes associated to peripheral neuropathy

Variable	*P* value
Fear of falling	0.001
Benzodiazepine use	0.042
Antihypertensive use	0.026
Deep tendon reflexes absence	0.049
Painful cold sensation	0.008
Fibular nerve altered sensibility	0.029
Neuropathic pain (DN4)	0.016
Balance (SPPB)	0.026

DAS-28_ESR_, Disease Activity Score -28—erythrocyte sedimentation rate; HAQ, Health Assessment Score; DN4, Neuropathic Pain Questionnaire; SPPB, short physical performance battery

Motor neuropathy, pure or combined with sensory neuropathy, was associated with longer times in the 5-repetition chair stand test (*P* = 0.049).

Peripheral neuropathy was not associated to diabetes (*P* = 0.133), disease duration (*P* = 0.697), disease activity (*P* = 0.879), functionality (*P* = 0.460), and to the number of falls at 12 months pre assessment (*P* = 0.350) and 6 months post assessment (*P* = 0.674).

The main individual data collected are shown in Table 4.

**Table 4:** Individual data collected

Patients	ENMG assessment	HAQ	Disease Activity*	SPPB	Pain in lower limbs	Deep tenn reflexes	Falls Retro.	Falls Prosp.
1	Sensorial neuropathy	Severe	Active	Poor	Neuropathyc	Absent	3	1
2	No alterations	Mild	Active	Mod	Nociceptive	Present	0	1
3	Motor neuropathy	Mod	Active	Poor	Neuropathic	Absent	5	1
4	No alterations	Mild	Active	Mod	Nociceptive	Present	0	0
5	No alterations	Severe	Active	Poor	Nociceptive	Present	0	0
6	Sens-Mot. neuropathy	Mod	Active	Poor	Neuropathic	Present	15	4
7	Sens-Mot. neuropathy	Mild	Active	Very poor	Neuropathic	Absent	1	5
8	Sens-Mot. neuropathy	Mild	Active	Mod	Absent	Absent	0	0
9	No alterations	Mild	Active	Mod	Neuropathic	Present	0	0
10	Sens-Mot. neuropathy	Severe	Active	Poor	Neuropathic	Absent	0	0
11	No alterations	Mild	Active	Good	Nociceptive	Present	0	0
12	No alterations	Mild	Active	Mod	Nociceptive	Absent	2	0
13	No alterations	Mod	Active	Mod	Nociceptive	Absent	6	2
14	Sens-Mot. neuropathy	Mild	Active	Mod	Absent	Absent	1	0
15	No alterations	Mod	Active	Mod	Nociceptive	Absent	0	0
16	No alterations	Mild	Active	Good	Neuropathic	Present	4	1
17	Sens-Mot. neuropathy	Mild	Active	Good	Nociceptive	Absent	1	0
18	Sens-Mot. neuropathy	Mod	Active	Mod	Absent	Present	2	0
19	Sensorial neuropathy	Mod	Active	Mod	Neuropathic	Present	3	0
20	No alterations	Mild	remission	Good	Nociceptive	Absent	5	2
21	No alterations	Mild	remission	Mod	Nociceptive	Present	0	0
22	Motor neuropathy	Mod	Active	Poor	Nociceptive	Present	2	2
23	No alterations	Mild	active	Mod	Nociceptive	Present	1	4
24	Sens-Mot. neuropathy	Mod	Active	Mod	Nociceptive	absent	4	7
25	No alterations	Mild	Active	Mod	Neuropathic	Present	0	0
26	No alterations	Mild	Active	Good	Nociceptive	Present	1	0
27	Sens-Mot. neuropathy	Mild	Active	Mod	Absent	Present	0	0
28	Sens-Mot. neuropathy	Mild	Active	Good	Nociceptive	absent	0	0
29	No alterations	Mod	Active	Mod	Absent	Present	0	0
30	Motor neuropathy	Mild	Active	Poor	Neuropathic	Absent	3	0
31	Motor neuropathy	Mild	Active	Good	Absent	Present	0	0
32	Sens-Mot. neuropathy	Mod	Active	Poor	Neuropathic	Absent	0	0
33	No alterations	Mod	Active	Mod	Nociceptive	Present	3	0

HAQ, Health Assessment Score; SPPB, short physical performance battery; *, Boolean remission criteria; Mod., moderate; Falls Retro., number of falls accessed retrospectively; Falls Prosp., number of falls accessed prospectively; Sens-Mot., sensory-motor

In the 12 months prior to the evaluation, 17 patients fell, totaling 62 episodes, only 6 patients reported more than 3 falls and 15 of them did not fall in the period. Falls occurred predominantly during the day (86.8%), at the patient's home (55.7%) being 73.5% inside the house and the others in the backyard, by tripping (59%) or slipping (21%). Most patients (85.4%) reported no injuries or minor injuries and only 6 falls led to hospital care, 4 of them for fracture.

The number of falls was significantly related to gender (*P* = 0.015), being higher in females, body mass index (r = 0.360, *P* = 0.040), use of antihypertensives (*P* = 0.007), and fear of falling (*P* = 0.030).

Fear of falling was higher in patients with neuropathic pain (*P* = 0.030) and with the associated symptoms of pinprick (*P* = 0.035) and painful cold sensation (*P* = 0.027).

Antidepressant was the unique drug significantly related to fear of falling (*P* = 0.014).

The SPPB showed a mean of 7.8 with a standard deviation of 2.21 and was worse among patients with diabetes mellitus (*P* = 0.001), neuropathic pain (*P* = 0.018), altered sural nerve sensitivity (*P* = 0.034), and those who used more than 4 medications (*P* = 0.045).

No relationship was found between balance (SPPB) and the number of falls in the previous 12 months.

In the six months following the evaluation 11 patients fell, totaling 30 episodes, and only 4 patients reported more than 3 falls. The falls occurred predominantly during the day (93.3%), in the patient's home (66.7%), being 76.7% inside the house and the others in the backyard, by tripping (43.3%) or slipping (30%). The majority (90%) reported no injury or minor injuries, 3 falls led to hospital care, and only one to fracture.

There was an association between falls in the previous 12 months and in the subsequent 6 months (*P* = 0.003); 55.6% of the patients who have fallen in the previous 12 months fell again in the subsequent 6 months.

The number of falls was significantly associated to BMI (R = 0.376, *P* = 0.031), but not to gender (*P* = 0.393), fear of falling (*P* = 0.117), antihypertensive (*P* = 0,168) and antidepressant use (*P* = 0.103), disease activity (*P* = 0.513), functionality, (*P* = 0,165) balance-(*P* = 0.145), and neuropathic pain (*P* = 0.871).

**Fig. 3:**
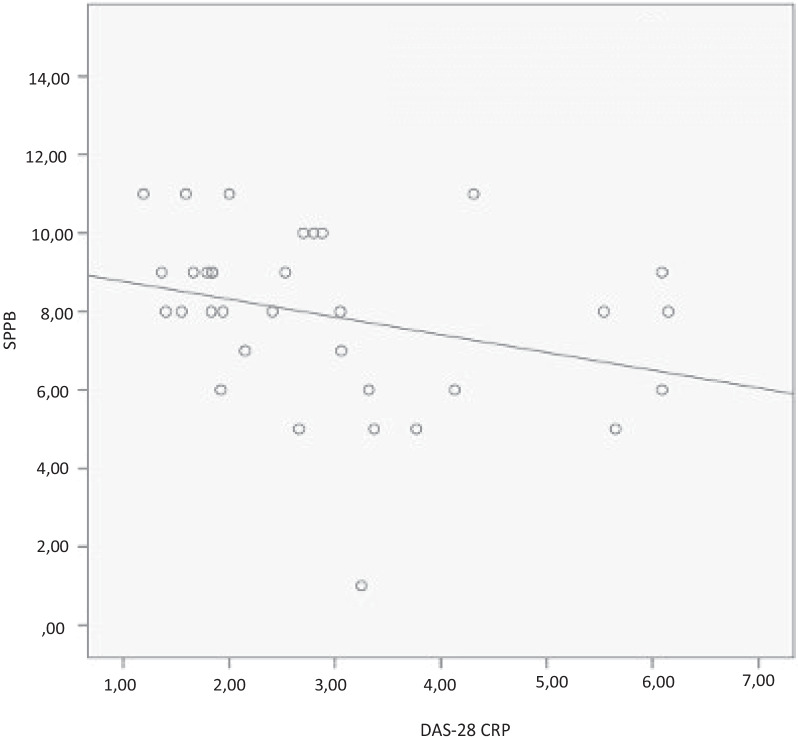
Correlation between balance and disease activity

**Fig. 4:**
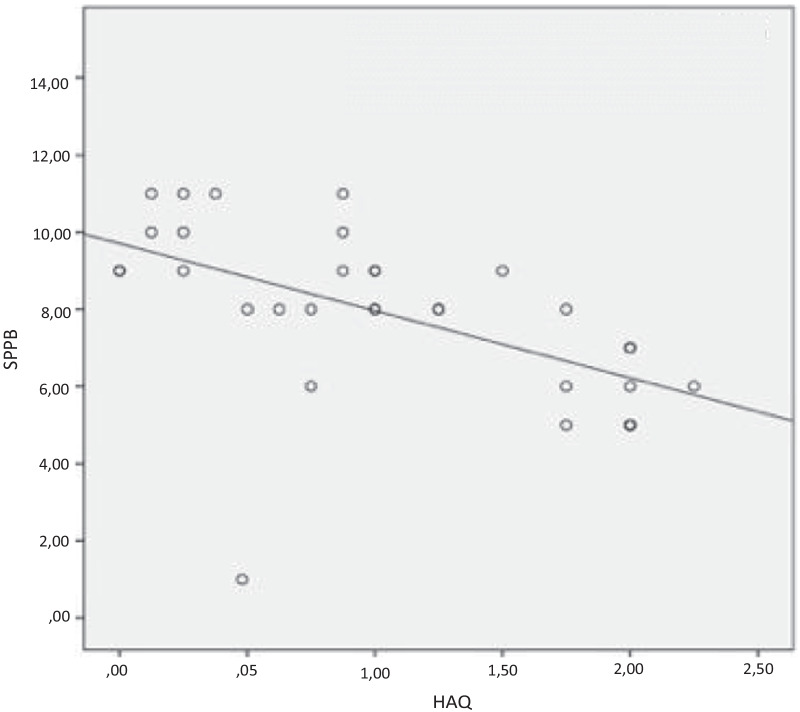
Correlation between balance and functionality

Patients with worse balance presented with higher disease activity with DAS-28_CRP_ (R = − 0.408, *P* = 0.018) and worse functionality (R = − 0.673, *P* = 0.000), as shown in Figs. 3 and 4.

## Discussion

The prevalence of peripheral neuropathy was 48.5%, in agreement with the literature, which presents results between 17 and 72.2% depending on the methodology and the local of assessment if in upper, lower limbs or both [13–16, 26].

The findings regarding the type of neural involvement are similar to the studies by Lanzillo et al. [27], Nadkar et al. [28], Bayrak et al. [13] and Sim et al. [2] in which there is a predominance of the sensorimotor pattern; however, they differ from Biswas et al. [26] and Agarwal et al. [29], who found a slight predominance of the sensory pattern in relation to the sensorimotor.

Regarding the type of neuronal lesion, the findings of the present study are like those in the literature in which there is a clear predominance of axonal type lesions, although it is difficult to compare due to differences in the methods of ENMG assessment [13, 14, 26, 27, 29].

There was no association between age, gender and peripheral neuropathy as well as in several previous reports [13, 15, 28], only the study by Sim et al. [2] found an association between age and peripheral neuropathy.

Most of the sample was women above 50 years old, which was also found in most studies due to two to three times higher prevalence of RA in females and common age of presentation starting from the fourth decade of life [30]. Although advanced, the age range was below the age in which alterations of the peripheral nerves are known to be detected, which is from 80 years on [31].

No previous study had investigated the association between neuropathy in patients with RA and the occurrence of falls. However, we did not find in our study a significant correlation between peripheral neuropathy and falls.

There was no significant association between neuropathy and disease activity indices which was also reported by Biswas et al. [26], Agarwal et al. [29] and Li et al. [32], different however from the results of Bayrak et al. [13] and Umay et al. [14], revealing that this is still a risk factor to be studied regarding neuropathy in patients with RA.

In the present study no association was found between peripheral neuropathy and functionality as well as in the studies by Agarwal et al. [29], Sim et al. [2] and Umay et al. [14], only the study by Bayrak et al. [13] found significant relationship between neuropathy and HAQ scores.

There was a significant association between peripheral neuropathy and balance disorders, which corroborates our hypothesis that neuropathy may contribute to the risk of falls, although it is not possible to establish a causal relationship in this type of study. The association between altered sural nerve sensation and balance disorder (*P* = 0.034) could also corroborate this hypothesis.

As expected, it was also observed association of neuropathic pain with peripheral neuropathy (*P* = 0.016) and neuropathy was found in 72.2% of patients with neuropathic pain as well as in the study of Filatova and Erdes [33] who found it in 96%.

Reduced cutaneous sensitivity in the fibular nerve was significantly associated to peripheral neuropathy as with Souza et al. [24] who demonstrated in patients with diabetes a good correlation between the severity of neuropathy assessed by monofilament examination and the ENMG exam. These findings help to support the hypothesis that the noninvasive monofilament test may be useful in the evaluation of peripheral neuropathy in patients with RA.

The absence of deep reflexes showed significant relation with peripheral neuropathy as well as in the studies of Good et al. [12] and Agarwal et al. [29], which was not confirmed by Yanshan et al. [32].

No other study was found on the relationship between peripheral neuropathy and fear of falling. In the present study, fear of falling was significantly associated with peripheral neuropathy (*P* = 0.001), suggesting that reduced muscle strength and altered sensitivity may increase walking instability and fear of falling.

Age in this study, showed a surprisingly negative correlation with the number of falls in the previous 12 months (r = − 0.375, *P* = 0.032) different from that found in the general population in which age is an important risk factor [7, 34]. Studies in RA found no relationship between age and falls [4, 33, 35]. We found a small negative association between age and falls, but the lower mobility of the older patients could be associated to a lower occurrence of falls. In the 6 months following the assessment, there was no association between the number of falls and age (R = − 0.340, *P* = 0.053).

The incidence of falls in the present study was 51.5% in retrospective analysis and 33.3% in 6-month prospective analysis agreeing with the literature that presents incidence from 10 to 54%, including two recent studies published 2019 and 2020 that found incidence of 52.2% and 51% respectively [36, 37].

Most of the falls, in both retrospective (55.7%) and prospective (66.7%) analysis occurred at home similarly to the study of Stanmore et al. (68.5%), Lourenço, Roma, and Assis [18], and Lourenço, Carli, and Assis [20]. It is likely that similarly to the elderly [7, 38], a combination of functional impairment and fear of falling reduces the patient's mobility to other environments, increasing the time staying at home where the environment is more familiar and safer.

Fear of falling was associated to the retrospective assessment of falls, as seen by Stanmore et al. [39] and Gaino et al. [36], but not in the prospective one as most of the studies [6, 35, 40, 41]. Few patients presented repeated falls in the retrospective and prospective assessments and that could be related to the post-fall syndrome in which a reduction of overall mobility and daily activities is observed after the first fall [42].

In the present sample there was a significant positive association between BMI and the occurrence of falls, both in the retrospective and prospective evaluation, although most of the studies have not found association [36, 41, 43], the largest cohort in the study by Furuya [44], confirmed our finding.

Most falls did not lead to serious consequences. In the 12 months prior to the assessment 17.6% of the group who fell had fractures, and 9.1% in the prospective evaluation, which agrees with the findings of most studies that have found a range between 5 and 18,3% [6, 43–47].

Most patients reported some type of lower limb pain, being 40.7% classified as neuropathic pain, but no association was found between the risk of falls or any type of pain, in retrospective and prospective evaluation, as in other studies [37, 44, 45]. Pain intensity measured by the visual analogue scale showed a positive relationship with falls in some studies [35, 45] but this data was not evaluated in our study.

We found an association between worse balance and neuropathic pain (R = 0.450, *P* = 0.018), but we did not find other similar study in patients with RA.

In the present study, patients with worse balance also had worse functionality. Assessment of balance and functionality in patients with RA was found only in the study by Aydog et al. [48] who assessed dynamic balance using the Biodex Stability System platform but found no differences in HAQ between patients who completed and those who failed to complete the test. Balance was also related to measures of disease activity DAS-28_ESR_, DAS-28_CRP_ and CDAI as well as in a previous study performed in our outpatient clinic and in the study by Bohler et al. [6, 49].

Association between balance and disease activity was not confirmed in The study by Dimachkie et al. [50] that used 4-m walk test and the study by Aydog et al. [48] that used dynamic platform balance test.

We found a medication use median of 6, higher than severe polypharmacy criterion proposed by Kusano [51], however, there was no relationship between the number of medications in use and the risk of falls in both retrospective and prospective evaluation, differing from the studies of Stanmore et al. [45] and Armstrong et al. [52].

Regarding use of antihypertensive drugs and the risk of falling the odds ratio observed were 7 in the present study, 9 in the study by Hayashibara et al. [43] and 2.82 in the study by Mikos et al. [37]. The mechanism to the risk increment risk in patients with RA is not known, but in elderly orthostatic hypotension has been suggested [53]. There was no elevation in the risk of falls in the prospective assessment.

The use of benzodiazepines and antidepressants that are often associated with the risk of falls in the elderly and in RA [7, 52] was not related to the number of falls in the present study, which may have been due to the small number of patients using these medications.

The duration of the disease had no correlation with the number of falls, as in several other studies [30, 35, 41].

Disease activity measures showed a sample with a predominance of disease in activity, with no correlation between this indexes and the number of falls evaluated retrospectively or prospectively, which agrees with several studies [30, 43, 44], but was not confirmed in other studies [36, 45, 49]. This shows that disease activity is still a risk factor to be studied in relation to falls in RA.

The functionality assessment showed a sample with mild disability and was not related to the number of falls in retrospective and prospective evaluation, as in several previous studies [35, 41, 43, 52]. Some recent studies found association, including Oh et al. [54] that evaluated a large cohort with 3469 patients [37].

We found no relationship between falls and altered balance in both retrospective and prospective evaluation, which is a controversial finding among studies even though the tests used to assess balance vary among them. The study by Lourenço, Roma, and Assis [6] showed moderate to strong correlation between SPPB and other balance tests such as the Berg Balance Scale and the Timed Up and Go Test. Kawabata et al. [55] in a study evaluating balance in patients with RA found no differences in SPPB between fallers and non-fallers, however there was a difference when only the 5-repetition chair stand test was used, suggesting that the scores in performance and/or balance could compensate the lower values of muscle strength. These findings are in line with our results, which showed longer times in the 5-repetition chair stand test in patients with any type of motor neuropathy, suggesting that motor impairment could be more important in increasing the risk of falls in patients with RA. Other finding that corroborates this hypothesis is that the major cause of falls in our sample was tripping, which can be explained by the motor impairment caused by the neuropathy leading to diminished foot dorsiflexion and hip flexion, impairments that are commonly associated to an increase in the trip related fall risk [56].

One limitation of our study is the small size of the sample, caused by the recruitment difficulties imposed by the COVID-19 pandemics, so we believe a study with bigger sample could help to find associations we did not find. Prospective studies with a control group could also help to stablish causal relation between neuropathy and the other outcomes, and studies comparing a group with intervention in muscle strength of lower limbs, with a control group, could also corroborate our finding that the motor impairment caused by the neuropathy could increase the risk of falls.

Another limitation of our study was not excluding patients diagnosed with diabetes, which is known to be the main condition associated with peripheral neuropathy [24]. This choice was made to maintain a larger sample size with better external validity, since diabetes is commonly found among patients with RA with incidence ranging between 9.2% and 20.7%, as demonstrated by a recent systematic review [57]. It must be considered that the peripheral nerve involvement caused by RA or diabetes are similar, since Good et al. [12] observed no differences in the electrophysiological study in RA in patients with and without diabetes.

The relevance of this study is evaluating, for the first time, the relationship between peripheral neuropathy, balance and falls in patients with RA. However, no association was found and the occurrence of falls in this group of patients still needs a better determination of risk factors and predictors.

## Conclusions

Peripheral neuropathy showed a high incidence in patients with RA and was associated with neuropathic pain, fear of falling, absence of tendon reflexes, alteration in the cutaneous sensitivity of the feet, and balance alteration.

Peripheral neuropathy was not associated with age, gender, disease activity, functionality, and with the occurrence of falls assessed retrospectively and prospectively.

The change in balance was associated with neuropathic pain and change in skin sensitivity of the feet, but not with the number of falls.

The occurrence of falls was associated with fear of falling, body mass index, and the use of antihypertensive drugs in retrospective evaluation and with body mass index in prospective evaluation. The occurrence of falls, in retrospective and prospective evaluation, was not associated with the duration of RA, disease activity, functionality, neuropathic pain, high number of medications, use of benzodiazepines and antidepressants.

Other studies, preferably with a larger sample, which evaluate peripheral neuropathy as a risk factor for falls in patients with RA are necessary to advance towards the knowledge of predictors and thus help prevent falls and its consequences.

## Data Availability

The datasets used and/or analyzed during the current study are available from the corresponding author on reasonable request.

## Abbreviations

RA: Rheumatoid arthritis
ENMG: Electroneuromyography
BMI: Body Mass Index
DAS-28: Disease Activity Score-28
ESR: Erythrocyte sedimentation rate
CRP: C-reactive protein
HAQ: Health Assessment Score
DN4: Neuropathic pain questionnaire
SPPB: Short physical performance battery
SD: Standard deviation
Min: Minimum
Max: Maximum
Kg: Kilogram
